# Service-learning and patient safety in medical education: a pretest–posttest study with a complementary expert perspective

**DOI:** 10.3389/fmed.2026.1948734

**Published:** 2026-10-14

**Authors:** Aracelly Núñez-Naranjo, Mauricio Carvache-Franco, Angeles Carrera-Criollo, Orly Carvache-Franco, Wilmer Carvache-Franco, Jaime Angamarca-Iguago

**Affiliations:** 1 Centro de Investigación en Empresa, Sociedad y Tecnología-ESTec, Facultad de Ciencias de la Educación, Universidad Tecnológica Indoamérica, Ambato, Ecuador; 2 Universidad Bolivariana del Ecuador, Durán, Ecuador; 3 Graduate School of Business, Universidad ESAN, Santiago de Surco, Lima, Peru; 4 Universidad Espíritu Santo, Samborondón, Ecuador; 5 Facultad de Ciencias Sociales y Humanísticas, Escuela Superior Politécnica del Litoral, ESPOL, Guayaquil, Ecuador; 6 Center for Evidence Ecosystems, Implementation Science, and Decision-Making (CIDES), Facultad de Ciencias de la Salud y Bienestar Humano, Universidad Tecnológica Indoamérica, Ambato, Ecuador

**Keywords:** home- and community-based services, older adults, perceived service availability, population aging, self-rated quality of life, sustainable development

## Abstract

**Introduction:**

Home- and community-based services (HCBS) may support quality of life among older adults when expected services are perceived as available. This study examined whether the number of expected HCBS perceived as available was associated with self-rated quality of life (QOL) contemporaneously and at the next scheduled survey wave.

**Methods:**

We analyzed longitudinal data from the Longitudinal Healthy Longevity Survey. Contemporaneous analyses included 24,180 person-wave observations from 8,699 individuals and used individual fixed-effects linear probability models with survey-year effects. Prospective analyses included 20,974 adjacent-wave pairs from 13,386 individuals and adjusted for baseline QOL, covariates, and origin-year effects. Sensitivity analyses examined alternative outcome scales, model specifications, interval length, service domains, and subgroup interactions.

**Results:**

In the contemporaneous model, one additional expected perceived as available was associated with a 0.00841 higher probability of good/very good QOL (95% CI: 0.00408–0.01275). In the baseline-adjusted prospective analysis, the coefficient was 0.00338 (*p* = 0.089), decreasing to 0.00094 (*p* =0.662) when restricted to three-year intervals. Prospective findings varied by model and outcome specification.

**Discussion:**

More expected HCBS perceived as available was associated with better contemporaneous self-rated QOL. Prospective evidence was weaker and sensitive to analytical specification. Findings should be interpreted as associations rather than causal effects and do not establish actual service use or objectively verified service provision.

## 1 Introduction

The training of health professionals faces the growing challenge of preparing students to perform safely and competently in complex clinical systems (1, 2), where errors do not usually derive solely from knowledge deficits but rather depend on failures in processes, communication, coordination, and decision-making in real practice contexts (3–5). In medical education, the development of technical competencies cannot be understood in isolation from understanding the system in which those competencies are exercised, particularly when student actions have indirect but critical implications for patient safety (1, 6).

More broadly, educational trajectories in higher education are shaped by interacting personal, academic, economic, family, and institutional factors, highlighting the importance of considering the wider educational context in which student learning and progression occur (7).

Several authors have noted that a significant proportion of adverse health events originates in the early stages of clinical processes, often invisible to students during their initial training (8–10). This raises a relevant educational challenge: how to design training experiences that enable students to understand the importance of their actions within complex systems and to assume professional responsibility from the earliest stages of their training?

Laboratory medicine constitutes a paradigmatic example of this educational challenge (11). The pre-analytical phase of the total testing process has consistently been identified as the most vulnerable segment of the diagnostic process, accounting for 60%–70% of clinical laboratory errors (6, 12). These errors are mainly related to failures in patient identification, prior preparation, sample collection, specimen handling and transport, as well as deficiencies in documentation and traceability (13, 14).

From an educational perspective, the pre-analytical phase represents a high-risk domain embedded in a sociotechnical system in which people, technologies, protocols, and organizational contexts interact (15, 16). Many pre-analytical activities are carried out beyond the laboratory's direct control and frequently involve students or trainees, increasing the likelihood of avoidable errors and underscoring the need for training that extends beyond procedural instruction to foster a systemic understanding of the diagnostic process (13).

In parallel, digital technologies are increasingly being incorporated into medical education to support interactive learning, formative assessment, timely feedback, and student engagement. Digital learning environments may complement experiential educational strategies by facilitating access to learning activities, recording student responses, and providing feedback that can support self-regulated learning and reflection (17–20). However, the educational contribution of digital technologies depends on how they are integrated into the broader pedagogical design rather than on the technology itself.

The available literature has predominantly focused on attitudinal or perceptual outcomes or on general clinical competencies such as empathy, social commitment, and understanding of the social determinants of health (21, 22). There is a scarcity of research that explicitly explores how Service-Learning contributes to the development of specific technical competencies in domains characterized by complex systems (17) and risks to patient safety, such as the pre-analytical phase of the clinical laboratory (23).

It is necessary to enable students to develop technical competencies in complex clinical domains through authentic participation in practice, integrating procedural learning with an understanding of patient safety and professional responsibility (24).

Although artificial intelligence has attracted increasing attention in medical education (18–20), not all automated digital functions constitute AI. In the present intervention, the digital component consisted of conventional platform features—interactive question delivery, response recording, automated correctness feedback, and performance reporting—embedded within the broader Service-Learning program. Accordingly, the study did not evaluate AI as an independent intervention component.

Service-Learning, grounded in principles of experiential learning, offers a pedagogical framework well suited to articulating technical training with real-world practice contexts and the social demands of the health system (25, 26). However, there is limited understanding of how it contributes to the learning of specific technical processes embedded in high-risk sociotechnical systems, such as the pre-analytical phase of the clinical laboratory.

The main objective of this study was to evaluate pre–post changes in medical students' self-reported familiarity, learning perceptions, and perceived competencies related to the pre-analytical phase of the clinical laboratory following participation in a Service-Learning intervention supported by digital tools.

## 2 Literature review

Contemporary theories of workplace learning maintain that professional competence develops primarily through participation in authentic practice rather than through decontextualized teaching of theoretical content (27). The Experience-Based Learning (ExBL) model conceives learning as an emergent process that results from the student's interaction with real tasks, professional tools, and communities of practice, mediated by structured supervision and reflection (28, 29).

Learning implies acquiring technical skills while at the same time developing professional judgment, identity, and a sense of responsibility through progressive participation in activities with real clinical meaning (29, 30). In contexts such as laboratory medicine, where pre-analytical errors may have relevant clinical consequences, ExBL offers a solid conceptual framework for designing educational experiences that connect technical learning with patient safety (31).

The pre-analytical phase of the Total Testing Process (TTP) has been consistently identified as the most vulnerable segment of laboratory medicine, concentrating the largest proportion of errors that compromise the validity of diagnostic results and patient safety (32, 33).

In health sciences education, Service-Learning has consolidated as an experiential methodology that integrates academic objectives with meaningful service to the community, promoting critical reflection, social responsibility, and professional identity development (21, 34, 35). Previous studies have shown that this strategy fosters meaningful learning and positive professional attitudes when incorporated into the curriculum in a structured manner (36–38). However, from the authors' perspective, little is known about how these frameworks operate when applied to highly technical domains such as laboratory medicine.

In this context, digital learning tools can complement experience-based learning by providing interactive activities, immediate automated feedback, response monitoring, and opportunities for continuous formative assessment. These functions may support active learning and reflection when embedded within a broader pedagogical strategy; however, their educational contribution should be interpreted in relation to the overall instructional design rather than attributed to the digital technology in isolation (39, 40).

## 3 Materials and methods

### 3.1 Study design

The study was structured following methodological recommendations for nonrandomized designs and the reporting criteria established by the TREND (Transparent Reporting of Evaluations with Nonrandomized Designs) guideline.

The study used a one-group quasi-experimental pretest–posttest design in which the quantitative component constituted the primary source of evidence. A semi-structured interview with one clinical laboratory expert was included as a complementary contextual perspective to support interpretation of the educational and patient-safety context. Because the expert component was based on a single informant, it was not treated as an independent qualitative strand and was not intended to provide qualitative saturation, triangulation, or independent confirmation of the quantitative findings.

The study was conducted at the Faculty of Medicine of the Indoamérica University (Ecuador) during the first academic period of September 2024–February 2025, within the framework of the Fundamentals of Clinical Laboratory course.

The intervention was implemented as part of the educational component associated with pre-analytical phase content (patient preparation, venipuncture, sample identification/labeling, transportation and preservation, and rejection criteria), in accordance with current approaches that recommend evaluating educational interventions in authentic practice environments and situated learning contexts (29, 30).

The eligible population consisted of students enrolled in the course during the study period. Convenience sampling was employed, as the intervention was implemented within a regular course and random assignment was not feasible due to logistical and academic considerations, a condition frequently observed in quasi-experimental studies in health sciences education (41, 42). The final sample comprised 34 students (64.71% female and 35.29% male) aged 19–23 years.

Students were included who: (a) had active enrollment in the course; (b) participated in the planned educational intervention; and (c) agreed to participate in the study through informed consent. Students who did not complete both measurements (pre-intervention and post-intervention) were excluded from the analysis to maintain within-subject comparability in the pretest–posttest design.

The intervention was based on the Service-Learning (SL) methodology applied to the pre-analytical phase of the clinical laboratory. It was implemented as a multicomponent educational program integrating theoretical instruction, supervised practical activities, community service experiences, structured reflection, and digitally supported formative assessment. The digital component used Kahoot! (Kahoot! ASA, Oslo, Norway) to deliver interactive formative questions, record student responses, provide immediate automated correctness feedback, and generate response reports for instructor review. The functions used in this study were conventional automated platform features; no AI-specific functionality was evaluated as an independent intervention component.

Students participated in structured sessions focused on patient identification, sample collection, biosafety, and professional responsibility, promoting the integration of theoretical knowledge, contextualized practice, community engagement, and interactive digital resources.

The study was conducted in accordance with the ethical principles for research involving human subjects established in the most recent version of the Declaration of Helsinki, which guarantees respect for the dignity, rights, safety, and well-being of human participants (43).

The research protocol was evaluated and approved by the Institutional Review Board (IRB) of the Indoamérica University (Ecuador) before data collection began. All participants received clear and sufficient information about the objectives, procedures, benefits, and potential risks of the study, and signed a written informed consent before their inclusion.

Participation was voluntary, with no academic incentives or coercion of any kind. The collected data were anonymized by assigning alphanumeric codes and handled confidentially, used exclusively for research purposes. The study also complied with the principles of confidentiality and data protection applicable to educational research in the health sciences. The dataset supporting the analysis presented in this study is documented in the research data record (45).

### 3.2 Pedagogical foundations and program components

#### 3.2.1 Theoretical module

This component included a systematic introduction to the pre-analytical phase of the clinical laboratory, covering patient preparation, venipuncture, sample identification and labeling, transportation, and sample reception. The content was aligned with the requirements of ISO 15189:2022, which establishes standards for quality and safety in clinical laboratory pre-analytical processes, as well as with international recommendations on the management of pre-analytical errors (12, 13).

#### 3.2.2 Supervised practical activities

Simulations of pre-analytical procedures were conducted using a standardized technical rubric as a structured formative observation and feedback tool. The rubric addressed patient identification, venipuncture technique, tube filling and homogenization, labeling, documentation, sample transport, biosafety, and professional responsibility. It was used to guide faculty observation and formative feedback during supervised practice. Rubric scores were not included in the analytic outcome dataset reported in this article, and no pre–post rubric analysis was performed. Accordingly, the quantitative findings reported in this study derive from the 11-item self-perception questionnaire and should not be interpreted as objective evidence of clinical performance.

### 3.3 Implementation phases

The educational intervention was designed as a Service-Learning model supported by digital environments and was implemented in five sequential phases. The program structure was grounded in the principles of experiential learning and supervised participation, consistent with Experience-Based Learning (ExBL), in which learning emerges through the interaction among the student, the task, the tools, and the real-world context. The evaluation and reporting of the study were planned in accordance with the TREND recommendations for nonrandomized intervention studies (41).

#### 3.3.1 Phase 1. Content selection and design of the formative Kahoot! quiz

Critical content related to the pre-analytical phase was identified based on its relevance to diagnostic quality and patient safety, including patient preparation, venipuncture, sample identification and labeling, sample handling and transportation, pre-analytical interferences, and causes of sample rejection. Based on these topics, a 10-item multiple-choice quiz was developed for formative use during the intervention through Kahoot!. This formative quiz was used to support applied discussion and immediate feedback and was distinct from the 11-item self-perception questionnaire used as the primary pre–post outcome measure described in Section 3.5. Scores from the 10-item formative quiz were not included in the primary or secondary pre–post statistical analyses (6, 12).

#### 3.3.2 Phase 2. Implementation of the formative quiz on a digital platform (formative gamification)

The 10-item formative quiz was administered using Kahoot! (Kahoot! ASA, Oslo, Norway). The platform delivered the questions, recorded responses, provided immediate automated correctness feedback, and generated descriptive response reports used by instructors to guide discussion and reinforcement. Response times of 20–60 s were configured according to item complexity. These were conventional automated platform functions; no AI-specific functionality was evaluated.

#### 3.3.3 Phase 3. In-person administration and guided participation

The activity was conducted face-to-face in the classroom, with screen projection and mobile device access. Individual participation and guided discussion were encouraged, with emphasis on justifying the correct answer based on technical pre-analytical phase criteria (including patient identification, tube selection, hemolysis management, sample volume, and transportation). This approach was designed to promote situated learning and context-dependent reasoning, recognizing that technical and professional competence is developed through interaction with tools, the environment, and professional practices (30).

#### 3.3.4 Phase 4. Structured feedback and conceptual reinforcement

Following the administration, an immediate analysis of the questions with the highest error rates was conducted based on reports generated by the digital platform. The structured feedback combined teacher interpretation with data from the system, enabling the identification of specific difficulties and reinforcement of critical content.

This approach integrated data-driven feedback with pedagogical guidance, promoting an understanding of the clinical relevance of pre-analytical events and their impact on patient safety (12, 14).

#### 3.3.5 Phase 5. Systematization of results and reflective closure

As a final step, the aggregated results (item-level correct response rates and overall performance) were documented, and a reflective debriefing was incorporated to strengthen professional responsibility and continuous improvement by linking technical learning with quality management. This closing phase aligned with the literature, which promotes the use of indicators and continuous improvement processes as key elements for reducing errors in the pre-analytical phase (13, 15).

Additionally, the use of digital tools allowed students to design educational resources supported by emerging technologies, strengthening digital competencies and fostering the transfer of knowledge to real contexts.

### 3.4 Community service with digital support

As a service component, students designed and delivered digital educational resources (infographics, videos, and virtual presentations) to reinforce good pre-analytical practices and patient safety (40). The use of student-created digital resources has been described as an effective strategy for consolidating learning, fostering reflection, and extending the educational impact to the broader community (40).

The intervention had a total duration of 16 weeks, comprising 48 h of theoretical instruction, 48 h of supervised practical simulation, and 48 h of community service activities. This structure is consistent with previous Service-Learning experiences in medical education that have reported significant educational benefits even in interventions of limited but intensive duration (37, 44).

### 3.5 Instruments

Quantitative data were collected using a structured 11-item self-perception questionnaire addressing satisfaction with learning strategies, familiarity with Service-Learning and the pre-analytical phase, perceived curricular alignment, theory–practice integration, awareness of social realities, collaborative participation, critical reflection, social responsibility-related perceptions, and respectful listening and tolerance.

Responses were recorded on a five-point Likert-type scale (1 = strongly disagree; 5 = strongly agree), with higher scores indicating more favorable self-reported perceptions. The questionnaire constituted the primary quantitative outcome measure. Because all 11 items were self-reported, questionnaire scores reflect participants' perceptions and do not provide a direct measure of objectively demonstrated knowledge, technical skill, or clinical performance.

For the primary within-participant analysis, an overall questionnaire score was calculated by summing responses across the 11 items, yielding a possible participant-level score ranging from 11 to 55. This aggregate score was used as a pragmatic omnibus summary of the participants' overall self-reported response profile across the questionnaire. Because the items address conceptually distinct educational constructs, the total score was not interpreted as a psychometrically validated unidimensional latent construct. Accordingly, its interpretation was complemented by item-level analyses and by exploratory descriptive conceptual item groupings.

The 11-item self-perception questionnaire underwent expert content review before its administration. The review focused on the clarity, relevance, coherence, and appropriateness of the items in relation to the educational objectives of the Service-Learning program and the pre-analytical clinical laboratory context. The available review documentation was qualitative rather than based on a standardized item-level numerical rating matrix; therefore, a formal quantitative content-validity coefficient, such as Aiken's V or the Content Validity Index (CVI), could not be calculated retrospectively. The expert documentation nevertheless records that observations were incorporated before the instrument was considered suitable for final administration.

Comparison of the documented questionnaire versions showed that the review process resulted primarily in minor wording and syntactic revisions intended to improve clarity and consistency. Items P2, P3, P4, P5, P6, P7, P9, and P10 underwent wording adjustments, whereas P1, P8, and P11 remained unchanged. No items were added or removed, and the original five-point Likert-type response scale was retained. The item-level wording changes are summarized in Supplementary Table S1.

Internal consistency of the questionnaire was evaluated separately for the pre-intervention and post-intervention administrations using Cronbach's alpha based on the participant-level responses to all 11 items. Additional item-level reliability diagnostics included corrected item-total correlations and Cronbach's alpha if item deleted. The corresponding results are reported in the Results section.

To provide a complementary expert perspective, a semi-structured interview was conducted with one clinical laboratory professional with experience in quality management and pre-analytical training. The interview explored the clinical relevance of the pre-analytical phase for patient safety, common errors observed in practice, perceptions regarding medical students' preparedness, and the potential educational relevance of experiential approaches such as Service-Learning. The interview was included to provide contextual interpretation only and was not designed or analyzed as an independent qualitative strand of the study.

### 3.6 Data collection procedure

Data collection followed a structured and sequential procedure comprising four stages: an introductory orientation session, pre-intervention assessment, implementation of the educational program, and post-intervention assessment.

Before the implementation of the Service-Learning program, students participated in a face-to-face introductory session that provided an overview of the study procedures, the principles of Service-Learning, and the relevance of the pre-analytical phase to patient safety. The session also allowed participants to clarify questions regarding their participation in the study.

Following this introductory session and before implementation of the 16-week educational program, participants completed the 11-item self-perception questionnaire described in Section 3.5. At the end of the program, the same questionnaire items and five-point Likert-type response scale were administered again, enabling paired within-participant comparison of responses across the two measurement occasions.

The mode of questionnaire administration differed between assessments: the pre-intervention questionnaire was administered in a face-to-face setting, whereas the post-intervention questionnaire was completed online. Thus, although questionnaire content and response scaling were maintained across measurement occasions, administration conditions were not equivalent. This procedural difference was considered when interpreting the pre–post findings and is acknowledged as a potential source of measurement bias in the Discussion.

### 3.7 Data analysis

Quantitative data were summarized using descriptive statistics, including means, standard deviations, and 95% confidence intervals, as appropriate.

The primary analysis evaluated the within-participant change in the overall 11-item questionnaire score from pre-intervention to post-intervention. The distribution of paired difference scores was assessed using the Shapiro–Wilk test and visual inspection of the participant-level paired difference-score distribution. Because the difference scores departed from normality, the Wilcoxon signed-rank test was used as the primary inferential comparison. The aggregate questionnaire score was treated as a pragmatic omnibus summary of the participants' overall self-reported response profile and not as a psychometrically validated unidimensional construct. A graphical presentation of the participant-level paired difference-score distribution is provided in Supplementary Figure S1.

As a sensitivity analysis, a paired-samples Student's *t*-test was performed to assess whether the inferential conclusion was consistent under a parametric paired-comparison approach. Effect size for the overall paired comparison was estimated using Cohen's dz, calculated as the mean within-participant difference divided by the standard deviation of the difference scores, and was reported with a 95% confidence interval. Values of approximately 0.2, 0.5, and 0.8 were interpreted as small, medium, and large standardized effects, respectively (46).

Exploratory item-level pre–post analyses were conducted using Wilcoxon signed-rank tests for each of the 11 five-point Likert-type items. To control the family-wise error rate across the 11 comparisons, *p*-values were adjusted using the Holm procedure. Adjusted two-sided *p*-values < 0.05 were considered statistically significant. Item-level mean differences were additionally reported as descriptive measures of the observed changes. Rankings based on these mean differences were interpreted descriptively and were not considered evidence that the magnitude of change differed statistically between items.

Internal consistency of the 11-item self-perception questionnaire was assessed separately for the pre-intervention and post-intervention administrations using Cronbach's alpha based on participant-level responses. Corrected item-total correlations and Cronbach's alpha if item deleted were also examined as item-level reliability diagnostics. Cronbach's alpha was interpreted as an indicator of internal consistency within the present sample and not as evidence of unidimensionality or structural validity, given that the questionnaire included items addressing several conceptually distinct educational dimensions (47).

For exploratory pedagogical interpretation, 10 of the 11 questionnaire items were organized into five conceptually related two-item groupings based on item content and their correspondence with the educational objectives of the intervention: theory–practice integration (P3, P5), experiential learning and critical reflection (P6, P8), participation and collaborative work (P7, P11), social responsibility (P9, P10), and satisfaction and curricular integration (P1, P4). Item P2, which assessed familiarity with the Service-Learning methodology, was analyzed separately and was not assigned to a conceptual grouping. These groupings were not derived from factor analysis or other structural-validation procedures and were therefore used solely for exploratory descriptive interpretation. They should not be interpreted as psychometrically validated subscales or latent dimensions, and no inferential comparisons were performed between conceptual groupings.

The complementary expert interview was analyzed descriptively rather than as an independent qualitative dataset. The transcript was reviewed to identify salient statements corresponding to the topics addressed in the semi-structured interview guide, including theory–practice relationships, technical and patient-safety considerations, professional responsibility, and the educational relevance of experiential learning. These observations were organized as a descriptive summary of the expert's perspective. Given that only one expert was interviewed, no claims of thematic saturation, qualitative representativeness, methodological triangulation, or independent corroboration were made. The expert perspective was used solely to provide contextual interpretation and was not used to validate or confirm the magnitude or statistical significance of the quantitative pre–post findings.

All statistical analyses were performed using SPSS version 26.0 (IBM Corp., Armonk, NY, USA). Statistical tests were two-sided, with a significance level of *p* < 0.05, except where *p*-values were adjusted for multiple comparisons as described above.

## 4 Results

### 4.1 Implementation of the service-learning intervention

As part of the presentation of the results, the implementation of the Service-Learning-based educational intervention carried out in an authentic clinical-teaching environment is visually documented. Figure 1 illustrates a face-to-face situated learning session in which students actively participated in educational activities related to the pre-analytical phase of the clinical laboratory, under faculty guidance and with institutional support.

**Figure 1:**
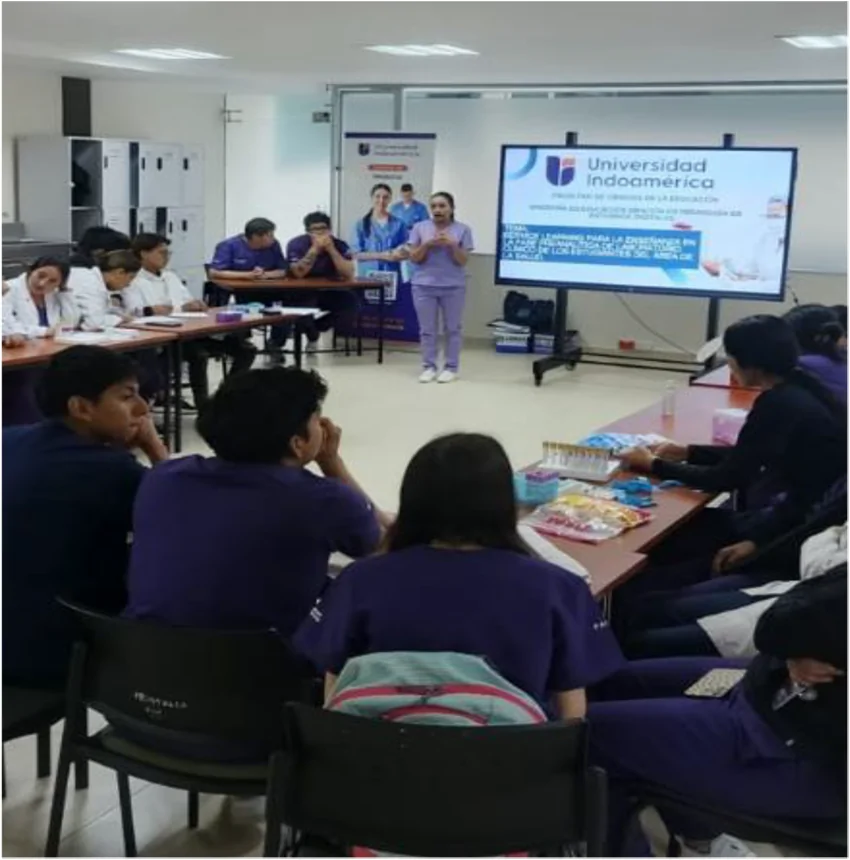
Implementation of the service-learning program among medical students during a face-to-face training session on the pre-analytical phase of the clinical laboratory.

The figure shows students' active participation in an authentic clinical-teaching environment, supported by faculty guidance and by institutional resources provided by the University.

#### 4.1.1 Supervised practical activities in the pre-analytical phase

Figure 2 documents the implementation of supervised practical activities in the pre-analytical phase of the clinical laboratory, specifically venipuncture procedures, in which students applied standardized technical protocols under faculty supervision, integrating theoretical knowledge with real clinical practice.

**Figure 2:**
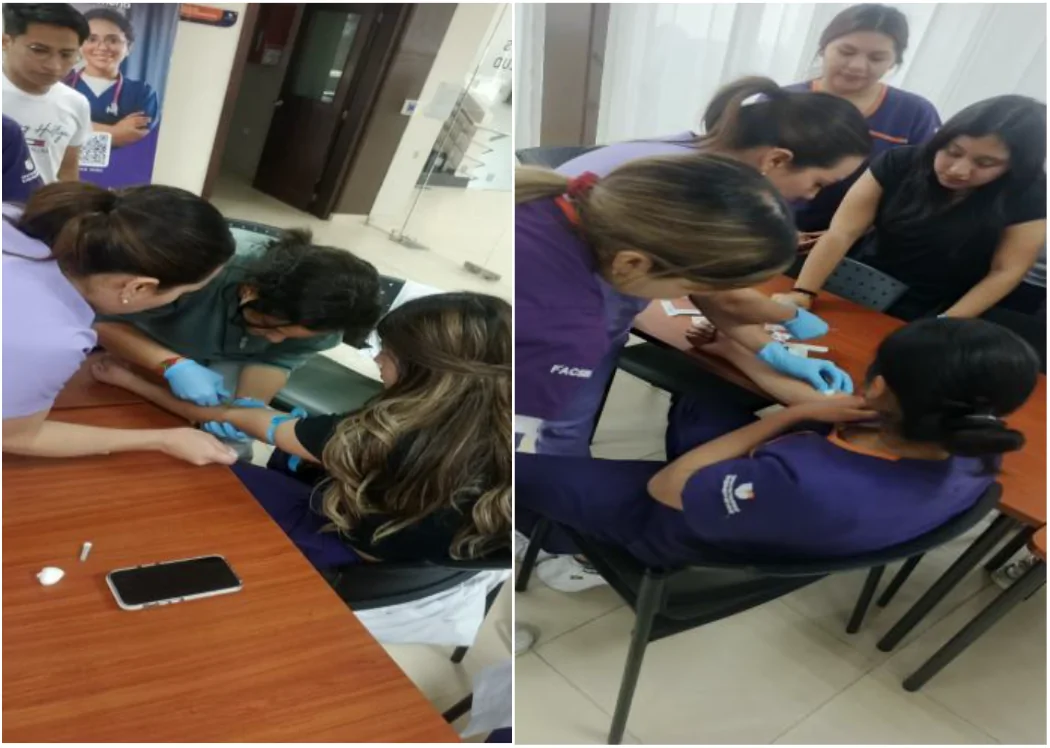
Development of supervised practical activities during the service-learning intervention.

The figure illustrates the performance of the venipuncture procedure during the pre-analytical phase of the clinical laboratory, with active student participation and faculty supervision in an authentic clinical-teaching environment.

#### 4.1.2 Community-based service implementation

As part of the community service component of the Service-Learning program, students performed pre-analytical procedures in real healthcare settings under faculty supervision, adhering to biosafety protocols and clinical laboratory technical standards. Figure 3 illustrates the performance of a venipuncture procedure in a community setting, demonstrating the integration of technical learning, social responsibility, and patient safety.

**Figure 3:**
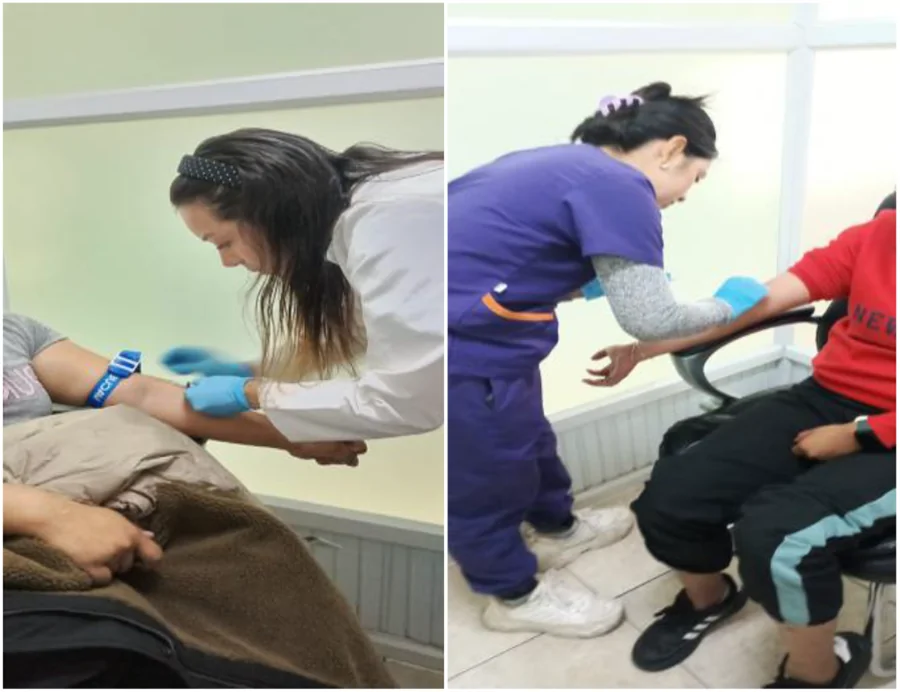
Community-based implementation of pre-analytical procedures during the service-learning intervention.

The figure shows a student performing a venipuncture procedure in a real community healthcare setting, under supervision and in accordance with biosafety protocols and pre-analytical quality standards.

### 4.2 Complementary expert perspective

The semi-structured interview with the clinical laboratory expert provided a complementary perspective on four aspects relevant to the educational context: the relationship between theory and practice, technical and patient-safety considerations, professional responsibility, and the educational relevance of experiential learning. These observations represent the perspective of a single informant and are presented solely to contextualize the quantitative findings.

Regarding theory–practice relationships, the expert emphasized the importance of opportunities that allow students to connect theoretical knowledge with activities situated in real healthcare contexts. From the expert's perspective, such exposure may help students recognize the pre-analytical phase as a critical component of the diagnostic process and patient safety.

Regarding technical and patient-safety considerations, the expert highlighted patient identification, venipuncture, sample labeling, and sample handling as areas in which supervised practical participation is particularly relevant. The expert also emphasized that errors during these processes may affect diagnostic quality and therefore require early attention during medical training.

Regarding professional responsibility, the expert emphasized responsibility, adherence to quality procedures, and awareness of the potential consequences of pre-analytical errors for diagnostic results and patient care.

The expert considered early exposure to real-world clinical contexts and experiential educational activities to be relevant for helping students connect technical processes with quality of care, patient safety, and interdisciplinary practice. These observations should be interpreted as contextual expert opinion rather than as independent qualitative evidence of intervention effectiveness.

### 4.3 Quantitative results

#### 4.3.1 Instrument content review and internal consistency

The expert content-review process resulted primarily in minor wording and syntactic revisions intended to improve item clarity and consistency. Based on the documented questionnaire versions, wording modifications were incorporated into P2, P3, P4, P5, P6, P7, P9, and P10, whereas P1, P8, and P11 remained unchanged. No items were eliminated or added, and the five-point Likert-type response scale was maintained. Because the original expert-review process did not retain a standardized item-level numerical rating matrix, a formal quantitative agreement coefficient such as Aiken's V or the Content Validity Index could not be calculated retrospectively. Therefore, the expert review is reported as evidence of content review rather than comprehensive psychometric validation.

Internal consistency was very high at both measurement occasions. Cronbach's alpha was *α* = 0.991 for the pre-intervention administration and *α* = 0.997 for the post-intervention administration. Corrected item-total correlations ranged from 0.930 to 0.971 at pre-intervention and from 0.972 to 0.987 at post-intervention. Cronbach's alpha if item deleted ranged from 0.990 to 0.991 in the pre-intervention assessment and from 0.996 to 0.997 in the post-intervention assessment, indicating that removal of any individual item did not meaningfully improve internal consistency. Given the very high alpha coefficients and the conceptually heterogeneous content of the questionnaire, these results are interpreted as evidence of strong internal consistency within the present sample and not as evidence of unidimensionality or structural validity.

Although these coefficients indicate very high internal consistency within the present sample, their magnitude should be interpreted cautiously. Values of this magnitude may reflect very strong inter-item covariance, possible item redundancy, or restricted response variability and may also be sample-specific given the relatively small cohort. Further psychometric evaluation in larger and more heterogeneous samples, including assessment of dimensionality and item redundancy, is warranted.

#### 4.3.2 Overall pre–post questionnaire results

The analysis of questionnaire responses showed higher post-intervention self-reported scores across the questionnaire items. The total score increased from 933 points in the pre-intervention assessment to 1,509 points in the post-intervention assessment. Similarly, the mean score per student increased from 27.44 to 44.38, representing a difference of 16.94 points (Table 1).

**Table 1:** Overall pre–post questionnaire results.

**Indicator**	**Pre-Intervention**	**Post-Intervention**	**Difference**
Number of students	34	34	—
Total score	933	1,509	+576
Mean score per student	27.44	44.38	+16.94

These results show higher post-intervention scores across the questionnaire items, including items addressing theory–practice integration, active participation, and broader professional-development perceptions. Given the one-group pretest–posttest design, these differences are interpreted as within-participant changes observed over the study period rather than as causal effects of the intervention.

#### 4.3.3 Descriptive results of the pre-intervention assessment

The distribution of responses obtained before the implementation of the educational strategy revealed heterogeneity in students' initial perceptions. Items addressing familiarity with Service-Learning and the pre-analytical phase showed a predominance of responses concentrated in the lower and intermediate levels of the scale.

Likewise, items related to the integration of theory and practice, active participation, and critical reflection showed substantial concentrations in the lower scale categories, indicating comparatively less favorable baseline self-reported perceptions in these areas (Table 2).

**Table 2:** Distribution of responses in the pre-intervention survey.

Item	1	2	3	4	5
P1. Satisfaction with learning strategies	9	9	7	6	3
P2. Familiarity with Service-Learning	4	10	7	7	6
P3. Familiarity with the pre-analytical phase	7	7	8	8	4
P4. Alignment with degree program objectives	11	5	6	9	3
P5. Relationship between theory and practice	16	7	5	4	2
P6. Identification of social realities	10	5	13	5	1
P7. Active and collaborative participation	16	6	5	5	2
P8. Critical reflection	9	14	2	8	1
P9. Social issues as learning opportunities	13	6	6	8	1
P10. Contribution to sustainable development	7	10	5	12	0
P11. Respectful listening and tolerance	18	4	3	5	4

#### 4.3.4 Post-intervention results

Post-intervention mean scores ranged from 3.88 to 4.12 and were numerically higher than the corresponding pre-intervention means across all questionnaire items.

The highest levels of agreement were observed for items addressing familiarity with Service-Learning, active and collaborative participation, theory–practice integration, and respectful listening and tolerance (Table 3).

**Table 3:** Descriptive results by item after the educational intervention.

ITEM	P1	P2	P3	P4	P5	P6	P7	P8	P9	P10	P11
Total Score	137	140	138	137	138	136	140	135	137	132	139
Mean	4.03	4.12	4.06	4.03	4.06	4.00	4.12	3.97	4.03	3.88	4.09

Five-point Likert scale (1 = strongly disagree; 5 = strongly agree).

#### 4.3.5 Pre–post intervention comparison

The overall questionnaire score increased from a pre-intervention mean of 27.44 to a post-intervention mean of 44.38, corresponding to a mean within-participant increase of 16.94 points.

Assessment of the distribution of the paired difference scores using the Shapiro–Wilk test indicated a statistically significant departure from normality (*W* = 0.905, *p* = 0.006). Consequently, the overall pre–post comparison was examined using the Wilcoxon signed-rank test, which showed a statistically significant difference between the two measurement occasions (*W* = 90.5, *p* < 0.001). A paired-samples Student's t-test conducted as a sensitivity analysis yielded the same inferential conclusion [*t*(33) = 4.18, *p* < 0.001]. The standardized paired effect size was Cohen's dz = 0.72 [95% CI (0.33, 1.09)], indicating a moderate-to-large standardized change. The observed distribution is shown in Supplementary Figure S1 and is consistent with the significant departure from normality identified by the Shapiro–Wilk test.

At the individual-item level, Wilcoxon signed-rank tests with Holm adjustment for 11 multiple comparisons indicated statistically significant pre–post differences for all questionnaire items (all Holm-adjusted *p* ≤ 0.015). Descriptively, mean increases ranged from +1.09 to +1.97 points. The numerically largest mean increases were observed for theory–practice integration (P5; Δ =  + 1.97), active and collaborative participation (P7; Δ =  + 1.97), and respectful listening and tolerance (P11; Δ =  + 1.88). These rankings refer only to the descriptive magnitude of the observed mean changes and should not be interpreted as evidence that the change in these items was statistically greater than that observed for the remaining items (Table 4).

**Table 4:** Pre–post comparison by questionnaire item.

Item	Pre mean	Post mean	*Δ*	Wilcoxon W	Holm-adjusted p
P1	2.56	4.03	+1.47	89.5	0.015
P2	3.03	4.12	+1.09	87.5	0.015
P3	2.85	4.06	+1.21	84.5	0.015
P4	2.74	4.03	+1.29	88.0	0.015
P5	2.09	4.06	+1.97	71.0	0.003
P6	2.47	4.00	+1.53	61.5	0.004
P7	2.15	4.12	+1.97	66.0	0.001
P8	2.35	3.97	+1.62	74.0	0.003
P9	2.35	4.03	+1.68	67.0	0.004
P10	2.65	3.88	+1.24	85.0	0.015
P11	2.21	4.09	+1.88	82.0	0.004

Δ = post-intervention mean minus pre-intervention mean. Item-level pre–post comparisons were performed using two-sided Wilcoxon signed-rank tests. *P*-values were adjusted across the 11 comparisons using the Holm procedure. Mean differences are presented descriptively and should not be interpreted as statistical comparisons of the magnitude of change between items.

These findings indicate that higher post-intervention self-reported scores were observed across items addressing familiarity with the pre-analytical phase, collaborative participation, critical reflection, and broader professional-development perceptions. These questionnaire-based differences should not be interpreted as evidence of improved objectively demonstrated knowledge, technical skill, or clinical performance.

#### 4.3.6 Items with the largest descriptive mean increases

For descriptive purposes, questionnaire items were ranked according to the magnitude of their observed pre–post mean differences. This ranking was intended to identify the items showing the largest numerical changes and does not constitute an inferential comparison of change magnitudes across items (Table 5).

**Table 5:** Questionnaire items with the largest descriptive pre–post mean increases.

Ranking	Questionnaire Item	*Δ*
1	Theory–practice integration (P5)	+1.97
1	Active and collaborative participation (P7)	+1.97
3	Respectful listening and tolerance (P11)	+1.88
4	Social issues as learning opportunities (P9)	+1.68
5	Critical reflection (P8)	+1.62

The largest descriptive mean increases were observed for theory–practice integration (P5; Δ =  + 1.97) and active and collaborative participation (P7; Δ =  + 1.97), followed by respectful listening and tolerance (P11; Δ =  + 1.88), social issues as learning opportunities (P9; *Δ* =  + 1.68), and critical reflection (P8; Δ =  + 1.62). These values describe the ordering of the observed changes; they do not demonstrate that the magnitude of change differed statistically between these items.

### 4.4 Exploratory descriptive analysis of conceptual item groupings

To provide an exploratory pedagogical summary of the response patterns, 10 questionnaire items were organized into five conceptually related two-item groupings, as described in the Methods section. These groupings were based on item content and the educational objectives of the intervention and were not derived from factor analysis or other structural-validation procedures. Therefore, they are presented as conceptual item groupings rather than validated questionnaire subscales, and the comparisons reported below are descriptive only (Table 6).

**Table 6:** Descriptive pre–post comparison by conceptual item grouping.

Conceptual item grouping	Items	Pre-intervention mean	Post-Intervention mean	*Δ*
Theory–Practice Integration	P3, P5	2.47	4.06	+1.59
Experiential Learning and Critical Reflection	P6, P8	2.41	3.99	+1.57
Participation and Collaborative Work	P7, P11	2.18	4.10	+1.93
Social Responsibility	P9, P10	2.50	3.96	+1.46
Satisfaction and Curricular Integration	P1, P4	2.65	4.03	+1.38

Group scores were calculated as the mean of the two items listed for each conceptual grouping. *Δ* represents the post-intervention minus pre-intervention mean change and was calculated using the underlying unrounded item-level means. Group means are displayed to two decimal places; therefore, *Δ* values may differ slightly from values obtained by subtracting the rounded means shown in the table. These groupings were created solely for exploratory pedagogical interpretation and were not derived from factor analysis or other structural-validation procedures; therefore, they should not be interpreted as psychometrically validated subscales or latent dimensions. No inferential comparisons were performed between conceptual groupings. Item P2 was analyzed separately and was not assigned to a conceptual grouping.

At the descriptive level, participation and collaborative work showed the largest numerical mean increase (Δ =  + 1.93), followed by theory–practice integration (Δ =  + 1.59) and experiential learning and critical reflection (Δ =  + 1.57). Social responsibility showed a mean increase of +1.46, whereas satisfaction and curricular integration showed an increase of +1.38. Because these conceptual item groupings have not undergone structural validation and no inferential comparisons of change magnitude were performed between groupings, these values should be interpreted solely as an exploratory descriptive summary of response patterns.

### 4.5 Contextualization of the quantitative findings with the expert perspective

The expert perspective was considered alongside the quantitative results only as contextual information. Several of the topics emphasized by the expert were conceptually related to areas addressed by the questionnaire. For example, the expert's emphasis on linking theoretical knowledge with real clinical activities was relevant to interpreting the higher post-intervention scores observed for theory–practice-related items. Similarly, the expert's comments regarding active participation, professional responsibility, and awareness of pre-analytical errors provided contextual background for interpreting questionnaire items addressing participation, reflection, and social responsibility, as well as the broader patient-safety context of the intervention.

These correspondences are presented as illustrative contextual links rather than as evidence of methodological triangulation or convergence. Because the contextual component was derived from a single expert interview, it cannot independently corroborate the quantitative findings, establish qualitative saturation, or strengthen causal inference.

### 4.6 Role of digital tools within the educational intervention

Kahoot! was incorporated as a supportive component of the multicomponent Service-Learning program. Its functions included delivery of interactive formative questions, immediate automated feedback, response recording, and generation of descriptive response reports that instructors used to guide subsequent feedback and discussion.

These digital functions were not analyzed as an independent intervention component. Because the program simultaneously included theoretical instruction, supervised simulation, community-based activities, structured reflection, and digital resources, the study design does not allow the contribution of Kahoot! or other individual components to be isolated.

Accordingly, the observed pre–post changes should be interpreted in relation to participation in the overall educational program rather than attributed independently to digital technology or artificial intelligence.

The digital platform therefore served as a delivery and formative-feedback mechanism within the broader educational intervention, and no AI-specific outcome or comparison was included in the study.

## 5 Discussion

The present study identified substantial pre–post differences in medical students' self-reported familiarity, learning perceptions, and perceived competencies related to the pre-analytical educational context following participation in a 16-week Service-Learning program supported by digital tools. Higher post-intervention scores were observed across the questionnaire items, including those related to theory–practice integration, collaborative participation, critical reflection, and broader professional-development perceptions. These findings indicate that changes occurred during the study period; however, because the study used a one-group pretest–posttest design without a comparison group, the observed changes cannot be attributed exclusively or causally to the intervention.

The initially moderate or low levels of familiarity with the pre-analytical phase are particularly relevant considering that this is one of the most vulnerable points in the laboratory process and is where errors that can compromise diagnostic quality and patient safety occur (32, 33). Along the same lines, Alcantara et al. (31) highlight the clinical importance of pre-analytical errors. This background, along with the initial results of the study, reinforces the need to incorporate educational experiences that allow for an early understanding of the relationship between laboratory procedures, error prevention, and their potential consequences for the patient.

Higher post-intervention self-reported scores were observed across the questionnaire items, particularly for items addressing theory–practice integration, active and collaborative participation, critical reflection, and socio-emotional aspects. These patterns are consistent with the broader educational literature suggesting that authentic and contextualized experiences may support professional learning (28, 29). Similarly, Koufidis et al. (30) describe the relevance of participation in practice-based scenarios for the development of professional judgment and responsibility. The present findings extend this discussion to the pre-analytical phase of the clinical laboratory; however, because of the uncontrolled pretest–posttest design, they should be interpreted as preliminary associations rather than evidence of intervention effectiveness.

The importance of participation and collaborative work aligns with the Service-Learning principles proposed by Furco (34) and Hu et al. (21), who highlight participation, reflection, and interaction with real-world situations as essential components of this methodology. Similarly, Mason and Dunens (37), Dubé et al. (36), and Ratnayake and Lederer (38) recognize that these experiences can foster meaningful learning and contribute to professional development.

The present response patterns extend this discussion to educational dimensions relevant to technically demanding clinical settings. In particular, higher post-intervention self-reported scores were observed for theory–practice integration, collaborative participation, critical reflection, and related professional-development perceptions. Given the uncontrolled design, these findings should be interpreted as preliminary within-cohort changes rather than as evidence that Service-Learning produced these outcomes.

The complementary expert perspective provided contextual observations relevant to the interpretation of the educational setting. The interviewed professional emphasized the importance of applying knowledge in real-world situations, recognizing the consequences of pre-analytical errors, and developing professional responsibility. These observations are conceptually consistent with the broader literature on authentic workplace learning (29, 30); however, because they derive from a single informant, they are presented solely as contextual expert opinion and should not be interpreted as qualitative corroboration or convergence with the quantitative findings.

From a patient safety perspective, West et al. (33), Iqbal et al. (32), and Alcantara et al. (31) have highlighted the vulnerability of pre-analytical processes and the consequences of errors occurring in this phase. While these studies primarily focus on identifying and analyzing such errors, the present research offers a complementary educational perspective. The observed response patterns are consistent with the educational relevance of contextualized experiences for linking pre-analytical practice with diagnostic quality and patient-safety concepts; however, the present design does not establish that the intervention caused such changes.

Digital tools complemented the educational process by supporting formative activities, automated feedback, interaction, and participation, consistent with previous research on game-based digital assessment in medical education (39, 40). However, these resources were embedded within a multicomponent program that simultaneously included theoretical instruction, supervised simulation, community-based service, and structured reflection. Therefore, the present study cannot determine the independent contribution of the digital component to the observed pre–post changes, nor can these changes be attributed specifically to artificial intelligence.

Several limitations should be considered when interpreting these findings. First, the one-group pretest–posttest design did not include a concurrent comparison or control group. Consequently, the observed pre–post differences cannot be attributed exclusively to the Service-Learning intervention, because alternative explanations for change cannot be ruled out. These may include maturation over the 16-week study period, exposure to other learning experiences within the academic course, regression to the mean, and other time-related influences occurring between measurements. Second, the same 11-item questionnaire was administered before and after the educational program. Repeated exposure to the same statements may have introduced a testing or retest effect, whereby familiarity with the questionnaire content or response process could have influenced post-intervention responses independently of substantive educational change. The present design does not allow the magnitude of this potential effect to be quantified. Third, the administration mode differed between measurement occasions. The pre-intervention questionnaire was administered in a face-to-face setting, whereas the post-intervention questionnaire was completed online. Although the same questionnaire items and five-point response scale were used, differences in administration context may influence response behavior, perceived anonymity, social desirability, or engagement with the questionnaire. This mode-of-administration difference therefore represents a potential source of measurement bias, and its direction and magnitude cannot be determined from the available data. Additional limitations include the relatively small sample, recruitment from a single institution, reliance predominantly on self-reported outcomes, and the absence of an external comparison group. Although a standardized technical rubric was used during supervised practical activities, rubric scores were used for formative observation and feedback and were not incorporated into the analytic dataset. Consequently, the present study cannot determine whether the observed changes in self-perception corresponded to objectively demonstrated improvements in pre-analytical knowledge, technical performance, or clinical competence. Together, these factors limit causal inference and generalizability. Future studies should incorporate controlled or comparative designs, standardize the mode of questionnaire administration across measurement occasions, consider alternative or parallel measurement forms when repeated testing is required, and include objective performance-based outcomes alongside self-reported measures.

A further measurement limitation concerns the psychometric status of the 11-item questionnaire. Although the instrument underwent expert content review and showed very high internal consistency in the present sample, no factor-analytic or other structural-validity assessment was conducted. Cronbach's alpha should therefore not be interpreted as evidence that the questionnaire represents a single latent construct. Likewise, the conceptual item groupings reported in this study were created solely for exploratory pedagogical interpretation and should not be interpreted as validated subscales. Future studies should evaluate the questionnaire in larger and more heterogeneous samples using formal structural-validity analyses and assessment of potential item redundancy.

The contextual expert component was also limited to a single clinical laboratory professional. Consequently, this interview cannot represent students' experiences or the range of perspectives of other stakeholders involved in the educational process and cannot support claims of qualitative saturation or transferability. Future studies seeking a substantive qualitative component should include students and multiple purposively selected stakeholders to capture variation in experiences and permit a more robust qualitative analysis.

Future research should incorporate larger samples, comparative groups, objective performance evaluations, and designs capable of estimating the contribution of specific components of multicomponent educational interventions, including digital tools.

A contribution of this study is the preliminary characterization of self-reported educational changes observed during a Service-Learning program focused on the pre-analytical phase of the clinical laboratory, an area for which educational evidence remains limited. The largest descriptive changes involved theory–practice integration, collaborative participation, reflection, and related professional-development perceptions. These observations identify educational dimensions that warrant further investigation in controlled studies rather than demonstrating intervention-specific effects.

The quantitative findings, considered alongside the complementary expert perspective, provide a broader contextual view of the educational process. The expert observations help situate the questionnaire findings within the clinical and patient-safety context but are not treated as an independent qualitative source of confirmatory evidence.

## 6 Conclusion

The present study identified statistically significant pre–post differences in medical students' self-reported familiarity, learning perceptions, and perceived competencies related to the pre-analytical phase of the clinical laboratory following participation in a Service-Learning program supported by digital learning technologies. Higher post-intervention scores were observed across the questionnaire items, including those addressing familiarity with the pre-analytical phase, theory–practice integration, collaborative participation, critical reflection, and broader professional-development perceptions.

The overall pre–post comparison showed a statistically significant within-participant difference accompanied by a moderate-to-large standardized effect size. These findings support the educational relevance of further investigating Service-Learning in this context; however, they should not be interpreted as evidence that the intervention itself caused the observed changes. The absence of a comparison group, repeated administration of the same questionnaire, and the difference between face-to-face pretest and online posttest administration preclude strong causal inference.

Accordingly, the findings should be interpreted as changes observed within this cohort over the study period and as preliminary evidence supporting further evaluation of this educational approach. Controlled studies using standardized administration procedures, objective performance outcomes, and larger multi-institutional samples are needed to determine the extent to which observed changes are specifically attributable to Service-Learning.

These findings contribute preliminary evidence regarding the application of Service-Learning in medical education, particularly in the relatively underexplored context of the pre-analytical phase of the clinical laboratory. The observed self-reported changes identify educational dimensions relevant to diagnostic quality, patient-safety education, and professional development that warrant further investigation. Digital tools served as supportive resources for formative feedback, participation, and learning activities within the broader educational program. Because these resources were not evaluated independently, no specific effect can be attributed to the digital component or to artificial intelligence.

Among the main limitations are the relatively small sample size, the conduct of the study within a single institution, the absence of a control group, and the predominant use of self-perception-based measures. Future research should incorporate experimental or controlled designs, larger samples, objective performance assessments, and analytical strategies capable of isolating the contribution of specific components, including digital functionalities, when this is a research objective.

The findings support continued investigation of Service-Learning supported by digital technologies as a potentially useful pedagogical approach for situated learning, professional development, and patient-safety education during the early stages of medical training. Confirmation of intervention-specific effects will require controlled quasi-experimental or experimental studies with appropriate comparison groups.

## Data Availability

The datasets presented in this study can be found in online repositories. The names of the repository/repositories and accession number(s) can be found in the article/Supplementary Material.

## Supplementary material

Supplementary Figure S1 Distribution of participant-level paired difference scores in the overall 11-item questionnaire score. Difference scores were calculated as post-intervention minus pre-intervention total questionnaire score for each of the 34 students. The distribution departed from normality (Shapiro–Wilk *W* = 0.905, *p* = 0.006), supporting the use of the Wilcoxon signed-rank test as the primary inferential comparison.

Supplementary Table S1 Documented item-level wording revisions of the 11-item questionnaire.

## References

[1] Dang TKC Park KH Lee ST. Health systems science and systems thinking: qualitative evaluation of preclinical medical student reflections in a role-play simulation game. BMC Med Educ. (2025) 25(1):1079. 10.1186/s12909-025-07594-w 40682036 PMC12275296

[2] Stevenson K D’Eon M Headrick L Gäre BA. A complex adaptive systems approach to health professions education transformation: the case of the university of Missouri-Columbia school of medicine and an integrated quality improvement-interprofessional collaborative practice curriculum. J Med Educ Curric Dev. (2025) 12:23821205251315624. 10.1177/23821205251315624 40027954 PMC11869308

[3] Gruppen LD Burkhardt JC Fitzgerald JT Funnell M Haftel HM Lypson ML. Competency-based education: programme design and challenges to implementation. Med Educ. (2016) 50(5):532–9. 10.1111/medu.12977 27072442

[4] Lee J-Y Huang C-H Wang H-W Hung S-W Chang J-T. A novel approach to patient safety education: integrating the human factors analysis and classification system (HFACS) to build a culture of safety in medical training. BMC Med Educ. (2025) 25(1):1296. 10.1186/s12909-025-07830-3 41039461 PMC12492519

[5] Van Der Vleuten CPM Schuwirth LWT. Assessing professional competence: from methods to programmes. Med Educ. (2005) 39(3):309–17. 10.1111/j.1365-2929.2005.02094.x 15733167

[6] Nordin N Ab Rahim SN Wan Omar WFA Zulkarnain S Sinha S Kumar S. Preanalytical errors in clinical laboratory testing at a glance: source and control measures. Cureus. (2024) 16:e57243. 10.7759/cureus.57243 38559530 PMC10981510

[7] Núñez-Naranjo AF. Analysis of the determinant factors in university dropout: a case study of Ecuador. Front Educ. (2024) 9:1444534. 10.3389/feduc.2024.1444534

[8] Lachman P Fitzsimons J. Transforming medical education to make patient safety part of the genome of a modern health care worker. JMIR Med Educ. (2025) 11:e68046. 10.2196/68046 39823210 PMC11758993

[9] Lippi G Chance JJ Church S Dazzi P Fontana R Giavarina D. Preanalytical quality improvement: from dream to reality. Clin Chem Lab Med. (2011) 49(7):1113–1126. 10.1515/CCLM.2011.600 21517699

[10] Plebani M Sciacovelli L Aita A Padoan A Chiozza ML. Quality indicators to detect pre-analytical errors in laboratory testing. Clin Chim Acta. (2014) 432:44–8. 10.1016/j.cca.2013.07.033 24012653

[11] Wei D Jiang Y Mao Y Xu Z Chen J Gao X. Phenyldivinylsulfonamides for the construction of antibody–drug conjugates with controlled four payloads. Bioorg Chem. (2023) 134:106463. 10.1016/j.bioorg.2023.106463 36924655

[12] Simundic A-M Lippi G. Preanalytical phase—a continuous challenge for laboratory professionals. Biochem Med (Zagreb). (2012) 22:145–9. 10.11613/BM.2012.017 22838180 PMC4062337

[13] Sciacovelli L Lippi G Sumarac Z Del Pino Castro IG Ivanov A De Guire V. Pre-analytical quality indicators in laboratory medicine: performance of laboratories participating in the IFCC working group “laboratory errors and patient safety” project. Clin Chim Acta. (2019) 497:35–40. 10.1016/j.cca.2019.07.007 31295446

[14] Tasneem A Zubair M Rasool Z Tareen FZ. Frequency and types of pre-analytical errors in a clinical laboratory of a specialized healthcare hospital. Pak J Med Sci. (2023) 40(2(ICON)):S70–S74. 10.12669/pjms.40.2(ICON).8963 PMC10844902 38328647

[15] Orhan B Sonmez D Cubukcu HC Zengi O Ozturk Emre H Cinaroglu I. The use of preanalytical quality indicators: a Turkish preliminary survey study. Clin Chem Lab Med (CCLM). (2021) 59(5):837–43. 10.1515/cclm-2020-1426 33079700

[16] Phelps M Yeoman P Monrouxe LV. Designing effective clinical education spaces: a scoping review. Med Educ. (2025) 59(12):1306–21. 10.1111/medu.70002 40844070 PMC12686777

[17] Hopson S Mildon C Hassard K Kubalek C Laverty L Urie P. Enhancing AI literacy in undergraduate pre-medical education through student associations: an educational intervention. BMC Med Educ. (2025) 25(1):999. 10.1186/s12909-025-07556-2 40611183 PMC12225195

[18] Topol EJ. Deep Medicine: How Artificial Intelligence Can Make Healthcare Human Again. New York: Basic Books (2019). Available online at: https://psnet.ahrq.gov/issue/deep-medicine-how-artificial-intelligence-can-make-healthcare-human-again (Accessed April 2, 2026).

[19] Masters K. Artificial intelligence in medical education. Med Teach. (2019) 41(9):976–80. 10.1080/0142159X.2019.1595557 31007106

[20] Wartman SA Combs CD. Medical education must move from the information age to the age of artificial intelligence. Acad Med. (2018) 93(8):1107–9. 10.1097/ACM.0000000000002044 29095704

[21] Hu C Nisbet G Chang Y. Responding to change in a medical student rural community service: insights from activity theory. Med Educ. (2022) 56(11):1086–95. 10.1111/medu.14869 35794821 PMC9796309

[22] Thackrah RD Hall M Fitzgerald K Thompson SC. Up close and real: living and learning in a remote community builds students’ cultural capabilities and understanding of health disparities. Int J Equity Health. (2017) 16(1):119. 10.1186/s12939-017-0615-x 28683808 PMC5500966

[23] Sepúlveda Maturana F Azocar González I González González ML Azocar González C Ramírez-Pereira M. The contribution of education to the correction of preanalytical errors in laboratory testing: a systematic review. Salud. Ciencia y Tecnología. (2025) 5:1781. 10.56294/saludcyt20251781

[24] Brydges R Butler D. A reflective analysis of medical education research on self-regulation in learning and practice. Med Educ. (2012) 46(1):71–9. 10.1111/j.1365-2923.2011.04100.x 22150198

[25] Sun T Xu X Zhu N Zhang J Ma Z Xie H. A service-learning project based on a community-oriented intelligent health promotion system for postgraduate nursing students: mixed methods study. JMIR Med Educ. (2023) 9:e52279. 10.2196/52279 38100207 PMC10757228

[26] Suresh A Wighton NM Sorensen TE Palladino TC Pinto-Powell RC. A hybrid educational approach to service learning: impact on student attitudes and readiness in working with medically underserved communities. Med Educ Online. (2022) 27(1):2122106. 10.1080/10872981.2022.2122106 36120928 PMC9518252

[27] Pavani M Bonaiuti P Chiroli E Gross F Natali F Macaluso F. Epistasis, aneuploidy, and functional mutations underlie evolution of resistance to induced microtubule depolymerization. EMBO J. (2021) 40(22):e108225. 10.15252/embj.2021108225 34605051 PMC8591535

[28] Billett S. Practice-based learning and professional education. In: Higgs J Barnett R Billett S Hutchings M Trede F, editors. Practice-Based Education. Rotterdam: SensePublishers (2012). p. 101–12. 10.1007/978-94-6209-128-3_8

[29] Dornan T Conn R Monaghan H Kearney G Gillespie H Bennett D. Experience based learning (ExBL): clinical teaching for the twenty-first century. Med Teach. (2019) 41(10):1098–105. 10.1080/0142159X.2019.1630730 31382787

[30] Koufidis C Manninen K Nieminen J Wohlin M Silén C. Representation, interaction and interpretation. Making sense of the context in clinical reasoning. Med Educ. (2022) 56(1):98–109. 10.1111/medu.14545 33932248

[31] Alcantara JC Alharbi B Almotairi Y Alam MJ Muddathir ARM Alshaghdali K. Analysis of preanalytical errors in a clinical chemistry laboratory: a 2-year study. Medicine (Baltimore). (2022) 101(27):e29853. 10.1097/MD.0000000000029853 35801773 PMC9259178

[32] Iqbal MS Tabassum A Arbaeen AF Qasem AH Elshemi AG Almasmoum H. Preanalytical errors in a hematology laboratory: an experience from a tertiary care center. Diagnostics. (2023) 13(4):591. 10.3390/diagnostics13040591 36832079 PMC9955620

[33] West J Atherton J Costelloe SJ Pourmahram G Stretton A Cornes M. Preanalytical errors in medical laboratories: a review of the available methodologies of data collection and analysis. Ann Clin Biochem Int J Lab Med. (2017) 54(1):14–9. 10.1177/0004563216669384 27614351

[34] Furco A. Service-learning: a balanced approach to experiential education. Expanding Boundaries: Serving and Learning. (1996) 1:1–6.

[35] Stewart T Wubbena ZC. A systematic review of service-learning in medical education: 1998–2012. Teach Learn Med. (2015) 27(2):115–22. 10.1080/10401334.2015.1011647 25893932

[36] Dubé TV Cumyn A Fourati M Chamberland M Hatcher S Landry M. Pathways, journeys and experiences: integrating curricular activities related to social accountability within an undergraduate medical curriculum. Med Educ. (2024) 58(5):556–65. 10.1111/medu.15260 37885341

[37] Mason MR Dunens E. Service-learning as a practical introduction to undergraduate public health: benefits for student outcomes and accreditation. Front Public Health. (2019) 7:63. 10.3389/fpubh.2019.00063 31001507 PMC6454065

[38] Ratnayake A Lederer A. Service learning in public health: a critical assessment of potential benefits and unintended consequences. Pedagogy Health Promot. (2024) 10(1):11–5. 10.1177/23733799231177043

[39] Ismail MA-A Ahmad A Mohammad JA-M Fakri NMRM Nor MZM Pa MNM. Using Kahoot! as a formative assessment tool in medical education: a phenomenological study. BMC Med Educ. (2019) 19(1):230. 10.1186/s12909-019-1658-z 31238926 PMC6593549

[40] Wang AI Tahir R. The effect of using Kahoot! for learning – A literature review. Comput Educ. (2020) 149:103818. 10.1016/j.compedu.2020.103818

[41] Des Jarlais DC Lyles C Crepaz N. Improving the reporting quality of nonrandomized evaluations of behavioral and public health interventions: the TREND statement. Am J Public Health. (2004) 94(3):361–6. 10.2105/AJPH.94.3.361 14998794 PMC1448256

[42] Harris AD McGregor JC Perencevich EN Furuno JP Zhu J Peterson DE. The use and interpretation of quasi-experimental studies in medical informatics. J Am Med Inform Assoc. (2006) 13(1):16–23. 10.1197/jamia.M1749 16221933 PMC1380192

[43] World Medical Association. World medical association declaration of Helsinki. JAMA. (2013) 310(20):2191. 10.1001/jama.2013.281053 24141714

[44] Chang BA Karin E Davidson ZA Ripp J Soriano RP. Impact of a short-term domestic service-learning program on medical student education. Ann Glob Health. (2019) 85(1):92. 10.5334/aogh.2465 31251483 PMC6634604

[45] Nuñez-Naranjo A. Dataset on the development of pre-analytical competencies through service-learning in patient safety education (1.0). (2026). 10.5281/zenodo.18452189

[46] Cohen J. Statistical Power Analysis for the Behavioral Sciences. Routledge (2013). 10.4324/9780203771587

[47] Tavakol M Dennick R. Making sense of Cronbach’s alpha. Int J Med Educ. (2011) 2:53–5. 10.5116/ijme.4dfb.8dfd 28029643 PMC4205511

