# Supplementary material for: Service-learning and patient safety in medical education: a pretest–posttest study with a complementary expert perspective
Source: Front Med (Lausanne). 2026 Oct 14;13:1948734. doi: 10.3389/fmed.2026.1948734 (PMC13713768; doi:10.3389/fmed.2026.1948734)
Supplement: Supplementary file 1 [file Datasheet1.docx]

**SUPPLEMENTARY MATERIAL**

Service-learning and patient safety in medical education: a pretest–posttest study with a complementary expert perspective

Frontiers in Medicine | DOI: 10.3389/fmed.2026.1948734 | Corresponding author: Aracelly Núñez-Naranjo

Reconstructed supporting tables based on the provided study workbook and manuscript. This is not a copy of the previously referenced but unavailable original Appendix A1–A11 / Notes S1–S3. All tables must be checked and authorized by the authors before submission.

# Supplementary Table A1. Questionnaire items and response coding

| **Item** | **Construct / item summary** |
| --- | --- |
| P1 | Satisfaction with learning strategies |
| P2 | Familiarity with Service-Learning |
| P3 | Familiarity with pre-analytical phase |
| P4 | Alignment with degree program objectives |
| P5 | Theory–practice integration |
| P6 | Identification of social realities |
| P7 | Active and collaborative participation |
| P8 | Critical reflection |
| P9 | Social issues as learning opportunities |
| P10 | Contribution to sustainable development |
| P11 | Respectful listening and tolerance |

Five-point response scale: 1 = strongly disagree; 5 = strongly agree, consistent with the main manuscript. The question wording is summarized here, not represented as verbatim participant-facing items.

# Supplementary Table A2. Baseline response frequencies

| **Item** | **1** | **2** | **3** | **4** | **5** | **N** |
| --- | --- | --- | --- | --- | --- | --- |
| P1 | 9 | 9 | 7 | 6 | 3 | 34 |
| P2 | 4 | 10 | 7 | 7 | 6 | 34 |
| P3 | 7 | 7 | 8 | 8 | 4 | 34 |
| P4 | 11 | 4 | 6 | 9 | 4 | 34 |
| P5 | 16 | 7 | 5 | 4 | 2 | 34 |
| P6 | 10 | 5 | 13 | 5 | 1 | 34 |
| P7 | 16 | 6 | 5 | 5 | 2 | 34 |
| P8 | 9 | 14 | 2 | 8 | 1 | 34 |
| P9 | 13 | 6 | 6 | 8 | 1 | 34 |
| P10 | 7 | 10 | 5 | 12 | 0 | 34 |
| P11 | 18 | 4 | 3 | 5 | 4 | 34 |

# Supplementary Table A3. Post-intervention response frequencies

| **Item** | **1** | **2** | **3** | **4** | **5** | **N** |
| --- | --- | --- | --- | --- | --- | --- |
| P1 | 3 | 4 | 4 | 1 | 22 | 34 |
| P2 | 2 | 2 | 5 | 6 | 19 | 34 |
| P3 | 3 | 3 | 4 | 3 | 21 | 34 |
| P4 | 2 | 5 | 3 | 4 | 20 | 34 |
| P5 | 3 | 3 | 4 | 3 | 21 | 34 |
| P6 | 2 | 4 | 5 | 4 | 19 | 34 |
| P7 | 2 | 2 | 5 | 6 | 19 | 34 |
| P8 | 2 | 4 | 5 | 5 | 18 | 34 |
| P9 | 2 | 5 | 4 | 2 | 21 | 34 |
| P10 | 4 | 3 | 4 | 5 | 18 | 34 |
| P11 | 3 | 4 | 3 | 1 | 23 | 34 |

# Supplementary Table A4. Item-level descriptive changes

| Item | Pre mean | Post mean | Difference |
| --- | --- | --- | --- |
| P1 | 2.56 | 4.03 | +1.47 |
| P2 | 3.03 | 4.12 | +1.09 |
| P3 | 2.85 | 4.06 | +1.21 |
| P4 | 2.74 | 4.03 | +1.29 |
| P5 | 2.09 | 4.06 | +1.97 |
| P6 | 2.47 | 4.00 | +1.53 |
| P7 | 2.15 | 4.12 | +1.97 |
| P8 | 2.35 | 3.97 | +1.62 |
| P9 | 2.35 | 4.03 | +1.68 |
| P10 | 2.65 | 3.88 | +1.24 |
| P11 | 2.21 | 4.09 | +1.88 |

# Supplementary Table A5. Paired overall questionnaire scores

| **Study ID** | **Pre total** | **Post total** | **Post − pre** |
| --- | --- | --- | --- |
| 1 | 18 | 55 | 37 |
| 2 | 11 | 35 | 24 |
| 3 | 37 | 55 | 18 |
| 4 | 12 | 24 | 12 |
| 5 | 45 | 54 | 9 |
| 6 | 26 | 55 | 29 |
| 7 | 11 | 11 | 0 |
| 8 | 41 | 55 | 14 |
| 9 | 19 | 46 | 27 |
| 10 | 14 | 55 | 41 |
| 11 | 29 | 33 | 4 |
| 12 | 12 | 55 | 43 |
| 13 | 54 | 11 | -43 |
| 14 | 17 | 54 | 37 |
| 15 | 11 | 55 | 44 |
| 16 | 42 | 28 | -14 |
| 17 | 23 | 55 | 32 |
| 18 | 37 | 39 | 2 |
| 19 | 12 | 55 | 43 |
| 20 | 51 | 17 | -34 |
| 21 | 29 | 50 | 21 |
| 22 | 16 | 55 | 39 |
| 23 | 21 | 55 | 34 |
| 24 | 49 | 21 | -28 |
| 25 | 14 | 55 | 41 |
| 26 | 35 | 45 | 10 |
| 27 | 11 | 55 | 44 |
| 28 | 45 | 24 | -21 |
| 29 | 25 | 55 | 30 |
| 30 | 18 | 55 | 37 |
| 31 | 28 | 49 | 21 |
| 32 | 31 | 33 | 2 |
| 33 | 41 | 55 | 14 |
| 34 | 48 | 55 | 7 |

Anonymized row identifiers reflect spreadsheet row order. Their correspondence across the two source sheets must be confirmed by the study team before considering this a verified paired-data table.

# Supplementary Table A6. Overall descriptive summary

| **Indicator** | **Baseline** | **Post** | **Change** |
| --- | --- | --- | --- |
| Students | 34 | 34 | — |
| Total questionnaire score | 933 | 1509 | 576 |
| Mean total score | 27.44 | 44.38 | +16.94 |
| Median total score | 25.5 | 54.5 | — |

# Supplementary Table A7. Primary inferential results reported in manuscript

| Measure | Reported result |
| --- | --- |
| Normality of paired differences | Shapiro–Wilk W = 0.905; p = 0.006 |
| Primary Wilcoxon signed-rank comparison | W = 90.5; p < 0.001 |
| Paired t-test sensitivity analysis | t(33) = 4.18; p < 0.001 |
| Paired standardized effect | Cohen’s dz = 0.72; 95% CI [0.33, 1.09] |

Values above are reproduced from the accepted manuscript; no independent statistical verification of the published inferential calculations is claimed.

# Supplementary Table A8. Item-level inferential outcomes reported in manuscript

| Item | Wilcoxon W | Holm-adjusted p |
| --- | --- | --- |
| P1 | 89.5 | 0.015 |
| P2 | 87.5 | 0.015 |
| P3 | 84.5 | 0.015 |
| P4 | 88 | 0.015 |
| P5 | 71 | 0.003 |
| P6 | 61.5 | 0.004 |
| P7 | 66 | 0.001 |
| P8 | 74 | 0.003 |
| P9 | 67 | 0.004 |
| P10 | 85 | 0.015 |
| P11 | 82 | 0.004 |

# Supplementary Table A9. Exploratory conceptual item groupings

| Grouping | Items | Pre | Post | Difference |
| --- | --- | --- | --- | --- |
| Theory–practice integration | P3, P5 | 2.47 | 4.06 | +1.59 |
| Experiential learning and critical reflection | P6, P8 | 2.41 | 3.99 | +1.57 |
| Participation and collaborative work | P7, P11 | 2.18 | 4.10 | +1.93 |
| Social responsibility | P9, P10 | 2.50 | 3.96 | +1.46 |
| Satisfaction and curricular integration | P1, P4 | 2.65 | 4.03 | +1.38 |

Descriptive groupings only; these have not been psychometrically validated as distinct subscales.

# Supplementary Table A10. Internal-consistency results from manuscript

| Indicator | Pre | Post |
| --- | --- | --- |
| Cronbach’s alpha | 0.991 | 0.997 |
| Corrected item–total correlation range | 0.930–0.971 | 0.972–0.987 |
| Alpha if item deleted | 0.990–0.991 | 0.996–0.997 |

Manuscript-reported results; these high values do not demonstrate unidimensionality.

# Supplementary Table A11. Intervention components and measured outcomes

| Component | Description |
| --- | --- |
| Theory | 48 hours of theoretical instruction |
| Supervised simulation | 48 hours of guided practical simulation |
| Community service | 48 hours of community activities |
| Program duration | 16 weeks |
| Main outcome | 11-item self-perception questionnaire before and after program |
| Additional context | One clinical laboratory expert interview; non-independent contextual perspective |
| Digital support | Kahoot! interactive questions and formative feedback; AI not evaluated independently |

# Supplementary Methods/Note S1. Study design and data provenance

One-group pretest–posttest quasi-experimental educational study among 34 medical students. Two spreadsheet worksheets (INITIAL_SURVEY and FINAL SURVEY) contain 34 rows each with eleven coded responses (P1–P11). Survey administrations differed: face-to-face before and online afterward. No control arm was included; no causal effect is established. Analysis assumes that respondent row IDs align between sheets. Confirm ID linkage before using the paired participant table or recomputing inferential statistics.

# Supplementary Methods/Note S2. Outcomes and analysis

Each item uses a 1-to-5 ordinal response scale with higher responses interpreted as more favorable perceptions according to the manuscript. The pragmatic overall score sums eleven items (possible range 11–55). Source manuscript reports the Shapiro–Wilk test for paired differences, Wilcoxon signed-rank primary analysis, paired t-test sensitivity analysis, standardized paired effect size Cohen’s dz, and Holm-adjusted item-level Wilcoxon analyses. Responses reflect student perceptions rather than objective competence or clinical performance. Extremely high Cronbach alpha values should not be treated as proof of a single latent factor.

# Supplementary Methods/Note S3. Supplementary content and interpretive limits

Tables A2–A6 and A9 are descriptive reconstructions from the source workbook; Tables A7, A8, and A10 reproduce main-manuscript statistics; A1 and A11 document study instruments and implementation. The source manuscript additionally identifies Supplementary Figure S1 and a distinct Supplementary Table S1 concerning item wording changes. Neither the underlying paired-difference graphic nor the original full A1–A11 / S1–S3 package was supplied as a separate verified archival supplement. Avoid publishing this reconstruction as if it were that original package. The authors should verify concordance, labels, source data and whether any table overlaps the article’s main tables.

**EDITORIAL STATUS: AUTHOR VALIDATION REQUIRED BEFORE UPLOAD.**
